# Pregnanolone Glutamate, a Novel Use-Dependent NMDA Receptor Inhibitor, Exerts Antidepressant-Like Properties in Animal Models

**DOI:** 10.3389/fnbeh.2014.00130

**Published:** 2014-04-16

**Authors:** Kristina Holubova, Tereza Nekovarova, Jana Pistovcakova, Alexandra Sulcova, Ales Stuchlík, Karel Vales

**Affiliations:** ^1^Institute of Physiology, Academy of Sciences of the Czech Republic, Prague, Czech Republic; ^2^Faculty of Medicine, Department of Pharmacology, Masaryk University, Brno, Czech Republic; ^3^Central European Institute of Technology, Masaryk University, Brno, Czech Republic

**Keywords:** depression, anxiety, NMDA channel blocker, neuroactive steroid, 3α5β-pregnanolone glutamate

## Abstract

A number of studies demonstrated a rapid onset of an antidepressant effect of non-competitive *N*-methyl-d-aspartic acid receptor (NMDAR) antagonists. Nonetheless, its therapeutic potential is rather limited, due to a high coincidence of negative side-effects. Therefore, the challenge seems to be in the development of NMDAR antagonists displaying antidepressant properties, and at the same time maintaining regular physiological function of the NMDAR. Previous results demonstrated that naturally occurring neurosteroid 3α5β-pregnanolone sulfate shows pronounced inhibitory action by a use-dependent mechanism on the tonically active NMDAR. The aim of the present experiments is to find out whether the treatment with pregnanolone 3αC derivatives affects behavioral response to chronic and acute stress in an animal model of depression. Adult male mice were used throughout the study. Repeated social defeat and forced swimming tests were used as animal models of depression. The effect of the drugs on the locomotor/exploratory activity in the open-field test was also tested together with an effect on anxiety in the elevated plus maze. Results showed that pregnanolone glutamate (PG) did not induce hyperlocomotion, whereas both dizocilpine and ketamine significantly increased spontaneous locomotor activity in the open field. In the elevated plus maze, PG displayed anxiolytic-like properties. In forced swimming, PG prolonged time to the first floating. Acute treatment of PG disinhibited suppressed locomotor activity in the repeatedly defeated group-housed mice. Aggressive behavior of isolated mice was reduced after the chronic 30-day administration of PG. PG showed antidepressant-like and anxiolytic-like properties in the used tests, with minimal side-effects. Since PG combines GABA_A_ receptor potentiation and use-dependent NMDAR inhibition, synthetic derivatives of neuroactive steroids present a promising strategy for the treatment of mood disorders.

**Highlights**:

- 3α5β-pregnanolone glutamate (PG) is a use-dependent antagonist of NMDA receptors.
- We demonstrated that PG did not induce significant hyperlocomotion.
- We showed that PG displayed anxiolytic-like and antidepressant-like properties.

## Introduction

Depressive disorders are among the most common and the most disabling mental diseases. There exist various and widely used antidepressants; however, one of their major limitations is a relatively long onset of antidepressant effect. But several studies demonstrate antidepressant properties of a single administration of ketamine – the non-competitive *N*-methyl-d-aspartic acid receptor (NMDAR) antagonist (Berman et al., 2000; Zarate et al., 2006, 2012; Diazgranados et al., 2010). Both subjective and objective evaluation of the mood after ketamine administration showed a significant improvement of the mood in the interval spanning from 2 h to 7 days (Entsuah et al., 2001; Thase et al., 2005). The minimum treatment of 2–4 weeks (often even more) is required to produce significant improvement in symptomatology with common antidepressants (Lam, 2012), while an infusion of ketamine to pharmaco-resistant patients show a similar effect (Murrough et al., 2013). The current hypothesis for the mechanism of ketamine action focuses on a complex cascade of neurochemical events that are induced by ketamine administration. These consequences persist for days after the ketamine elimination. First of all, ketamine administration blocks NMDAR. In all the cases, the protracted antidepressant effect is mediated by the consequent neuroplastic alterations (see Browne and Lucki, 2013; Hayley and Litteljohn, 2014).

It has been more than 20 years since the first proof of antidepressant action of the NMDAR antagonist emerged (Trullas and Skolnick, 1990). Since then, a growing number of evidence confirms that glutamate neurotransmission plays a crucial role in the neuropathology of the depression. Researchers have found that various types of drugs impairing NMDAR functioning (competitive, non-competitive and uncompetitive antagonists, and allosteric modulators) display antidepressant effects in the preclinical (Layer et al., 1995; Rogóz et al., 2002; Li et al., 2011; Burgdorf et al., 2013; Lapidus et al., 2013; Pilc et al., 2013) as well as in the clinical trials (Zarate et al., 2006, 2012). However, the clinical use of NMDA antagonists in pharmacotherapy of mood disorders is hampered by severe side-effects, particularly by psychotic symptoms in humans (Krystal et al., 1994). For this reason, research of the NMDA antagonists is a prominent topic in current neurobiology of the depressive disorder. It focuses on the elucidation of mechanisms of their antidepressant effect, and on the development of novel antidepressants – drugs with antidepressant properties and minimal side-effects, i.e., with more favorable benefit/risk ratio.

Therefore, the research and development of the novel therapeutics based on the NMDA antagonists is necessary in order to avoid psychotomimetic effects. These negative behavioral effects are most pronounced in the case of non-competitive antagonists. Conversely, the behavioral side-effects of the uncompetitive antagonists (antagonists selective for NMDAR containing a NR2B subunit or NMDAR glycine binding site antagonists) are less severe (Danysz et al., 1998; Popik et al., 1998; Karcz-Kubicha et al., 1999; Parsons, 2001; Kemp and McKernan, 2002; Chen and Lipton, 2006).

Neurosteroids are involved in several CNS physiological and pathological processes, such as the response to stress, depression, anxiety, sleep, or memory deficit (see more Morrow, 2007). Antidepressant effects of neuroactive steroids were described in animal models (Urani et al., 2001) as well as in patients (Wolkowitz et al., 1997, 1999). It has been shown that antidepressant treatment normalized the imbalance of 3α and 3β pregnanolone in patients suffering from depression (Romeo et al., 1998; Schüle et al., 2011). During social isolation, an animal model of depression-like behavior, biosynthesis of pregnanolone is significantly decreased (Pinna et al., 2008). SSRI are able to reverse the decreased brain pregnanolone level, and to correct behavioral deficits (Pinna et al., 2006).

The neurosteroids are known for their potentiation as well as inhibition of NMDA and GABA receptors. Naturally occurring 3α5β-pregnanolone sulfate has a substantial inhibitory activity (Irwin et al., 1994; Weaver et al., 2000; Kussius et al., 2009) on tonically activated NMDAR (Petrovic et al., 2005). Therefore, we introduced the development and testing of a novel synthetic NMDA antagonists derived from the 3αC pregnanolone having improved pharmacokinetic properties (Rambousek et al., 2011).

The newly synthesized neuroactive steroid 3α5β-pregnanolone glutamate (PG) is a representative member of a group of the steroids exerting effects on GABA_A_, AMPA, kainate, and NMDAR. Concerning the NMDAR, the mechanism of an action is not fully understood in detail, but it can be stated that it displays specific properties. The drug is an allosteric inhibitor of the NMDAR. Its degree of inhibition of the NMDAR currents is independent of the cell membrane potential. On the other hand, the binding to its inhibitory binding site is pre-conditioned by the activation of NMDAR by agonists. Therefore, it is so-called a use-dependent allosteric inhibitor of NMDAR (see Korinek et al., 2011) with more potent inhibition of responses mediated by NR1/NR2C-D receptors, compared to those mediated by the NR1/NR2A-B receptors (Petrovic et al., 2005) and GABA_A_ agonist (unpublished data). On the contrary to the non-competitive NMDA antagonists, 3α5β-PG is devoid of its adverse side-effects. 3α5β-PG binds only to the extrasynaptic and tonically activated NMDAR, which results in use-dependent selectivity (Rambousek et al., 2011).

In the present study, we examined potential antidepressant activity of PG. The effect of a single dose administration of PG was assessed by the Porsolt forced swim test, and by the repeated stress of social defeat, both used as common animal models of depression. The locomotor activity and the anxiolytic properties of PG were evaluated together with an open field and elevated plus maze tests. The effect of the chronic administration of PG on the aggressive behavior of singly housed male mice was evaluated by paired agonistic interactions with the non-aggressive group-housed partners.

## Materials and Methods

### Animals

#### Open field, elevated plus maze, and forced swimming studies

Naive adult male ICR mice (VELAZ s.r.o., Prague, Czech Republic), 15 weeks old and weighing 25–35 g, were used for the experiments. The animals were housed in groups of five in the plastic cages in a keeping of Institute of Physiology, Academy of Sciences of the Czech Republic. The mice had *ad libitum* access to the laboratory chow and water, except during behavioral experiments, and they were kept in a regulated environment (22°C, 50% humidity) under a 12-h light/dark cycle (lights on at 06:00 a.m.).

#### Social defeat and agonistic interaction studies

Naive adult male mice (ICR strain, VELAZ s.r.o., Prague, Czech Republic, 30–37 g) were used in this study. Food and water were available *ad libitum*. Mice were housed in a keeping of Department of Pharmacology, Faculty of Medicine of Masaryk University, Brno, either individually without any handling in self-cleaning cages with a grid floor (8 cm × 6 cm × 13 cm), or in groups of 17–20 in standard plastic cages (38 cm × 22 cm × 14 cm) with the floors covered with wooden shavings. The animals were housed, and behavioral testing was performed in a different room during the light phase of the constant light–dark cycle, with lights on at 06:00 and off at 18:00 h. The temperature was maintained at 21°C, and relative humidity was 50%. The group-housed mice were not handled, except on the experimental days. Singly housed mice were handled after 3 weeks of isolation, just during oral administration of PG.

Experiments were carried out between 09:00 a.m. and 06:00 p.m. All animal procedures were conducted in accordance with the European Community Council Directives of November 24, 1986 (86-609/EEC), and the Decree of October 20, 1987 (87–848). The study protocol was approved by the Animal Care Committee of the Institute of Physiology of Academy of Sciences of the Czech Republic and Masaryk University Brno, Faculty of Medicine, Czech Republic.

### Drug administration

Drugs and chemicals used in the study were purchased from Sigma-Aldrich (Germany). PG was prepared by the esterification of 3α-hydroxy-5β-pregnan-20-one (Steraloids Inc., USA) with a protected glutamic acid. The synthesis is thoroughly described in Rambousek et al. (2011). PG solutions were prepared by dissolution of PG in hydroxypropyl-β-cyclodextrin (β-CD, 72 mM saline solution, pH adjusted to 7.4 by 1 M NaOH). MK-801 and ketamine were prepared by dissolution in saline. β-CD was administered as a control for PG, and saline as a control for dizocilpine (MK-801) and ketamine. Dizocilpine was used as a representative of non-competitive highly selective NMDA antagonists. In addition, ketamine was used as a non-competitive NMDA antagonist possessing antidepressant activity. In the open-field test, PG was applied i.p. at doses of 0.1, 1, and 10 mg/kg, and dizocilpine at the dose of 0.3 mg/kg, and ketamine at the dose of 10 mg/kg. In the elevated plus maze test, PG was injected i.p. at doses of 1 and 10 mg/kg. In the forced swim test, PG was administered i.p. at doses of 0.1, 1, and 10 mg/kg, and in the social defeat test and agonistic interactions orally at the dose of 1 mg/kg via gastric tube. In these tests, PG was administered 30 min prior to behavioral testing at a volume of 1 ml/kg, except for the social defeat test and agonistic interactions, where PG was administered 60 min prior to testing. In agonistic interactions, PG at the dose of 1 mg/kg/day was administered orally once daily for 30 days at a volume of 1 ml/kg. All animals received the same volume of liquid per 1 kg of body weight.

### Behavioral procedures

#### Open-field test

**Figure 1:**
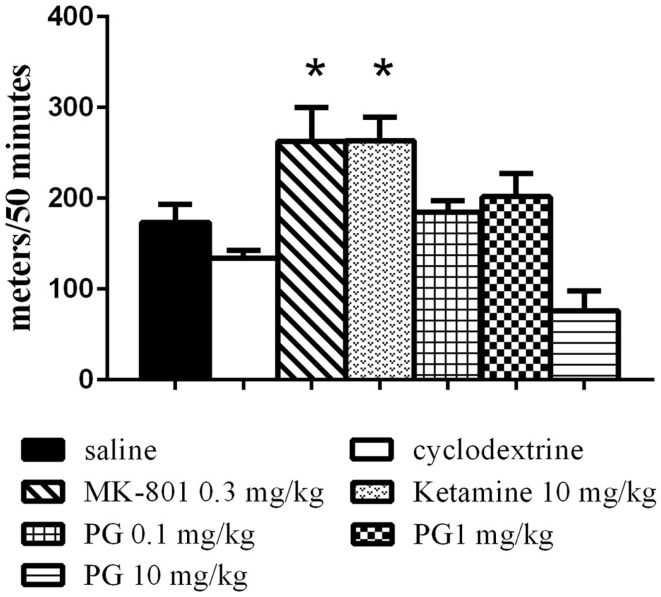
**Locomotor activity in the open field was assessed by placing an animal in the arena immediately after i.p. application of PG, MK-801, ketamine, and their vehicles**. Locomotor activity was monitored over 50 min. Ketamine and dizocilpine administration resulted in hyperlocomotion. Animals treated with these non-competitive NMDA antagonists significantly increased distance traveled. PG administration did not lead to significant changes in locomotion. All values represent group means ± SEM. *p < 0.05 compared to the saline group.

The animals were placed individually into a circular open-field arena (82 cm in diameter), located in a soundproof room. The locomotor activity in the open field was assessed by placing the animal in the arena immediately after i.p. application of PG, MK-801, ketamine, and their vehicles, and monitoring their activity over 50 min using a video tracking system (iTrack, Biosignal Group, USA). We analyzed the locomotor activity, expressed as a total distance traveled (Figure 1). The number of animals per group was as follows: five animals in ketamine, MK-801 and PG 10 mg/kg group, seven mice in PG 0.1 mg/kg group, eight mice in saline and PG 1 mg/kg group, and nine mice in β-CD group.

#### Elevated plus maze

The apparatus consisted of open arms (30 cm × 6.5 cm), crossed at right angles, with two arms of the same length enclosed by walls of 15.5 cm high (closed arms). The whole apparatus was raised 50 cm above the floor. At the beginning of each session, the mouse was placed on the central platform (6.5 cm × 6.5 cm) facing the closed arm. The time spent in the open, close arms, and central platform was recorded over a 10-min test session by a video recorder positioned above the maze, tracked, and analyzed by software (iTrack, Biosignal Group, USA). Each experimental group consisted of 10 mice.

#### Forced swimming

The forced swimming test was carried out in accordance with the methods described by Porsolt et al. (1978), only with slight modifications. Mice were randomly assigned into the three groups – vehicle treated (10 mice), PG 0.1 mg/kg treated (10 mice), PG 1 mg/kg (8 mice), and PG 10 mg/kg (8 mice) treated mice. Each mouse was placed in a 25-cm plastic transparent cylinder (12 cm in diameter) containing 10 cm of water at 24 ± 1°C. The mice were left in the cylinder for 6 min, and their behavior was recorded. The duration of immobility was scored in the last 4 min of the swimming test. Mice were considered to be immobile if they floated while making only necessary movements to keep the head above water (Porsolt et al., 1978). In addition to immobility duration, we analyzed time to the first floating. Latency was measured immediately after the mice were put into the water. Recorded videos were analyzed by two independent observers, blind to the treatment conditions. Analysis was performed by Observer 3.0 (Noldus Information Technology, The Netherlands) software, and the results were displayed as ethograms in excel tables.

#### Social defeat

The chronic social defeat stress procedure was carried out using a similar method, described by Sulcova and Krsiak (1987) and Pistovcakova et al. (2005). In the first part of the experiment, mice were given β-CD (14 mice) or PG (18 mice) orally via a gastric tube. Administration was carried out in a randomized order, 60 min prior to the open-field test observations performed in the identical animal cage, but in a different room from that used for the social defeat interactions. Each animal was placed singly into the center of a novel environment (arena 30 cm × 30 cm) of the PC-controlled tracking apparatus Acti-track (Panlab, S.L., Spain) with the infrared beam sensors. Over the 8-min testing period, the overall distance traveled (as a marker of locomotor/exploratory behavior) in the open field was measured. Two days later, each mouse was defeated with a singly housed mouse exhibiting an aggressive behavior in a 4-min paired agonistic interaction. The procedure was repeated four times, 7 days apart. Immediately after the last (fourth) agonistic interaction, each mouse was randomly assigned to the vehicle (12 mice), or the treatment group (6 mice). Sixty minutes following the drug administration, the animal was placed into the open-field arena, and the overall distance traveled was measured, as described above. Mice that received a timid partner instead of an aggressive one were excluded from the experiment, since they were not defeated.

#### Agonistic interactions

Prior to the experiment, mice were housed individually for 3 weeks. Each individually singly housed mouse was allowed 30 min adaptation in a Plexiglas neutral observation cage (20 cm × 20 cm × 30 cm) with clean wood shavings before it was coupled with a group-housed non-aggressive male partner for 4 min interaction. On the first day of the experiment, each animal received vehicle orally via a gastric tube 60 min prior to the agonistic interaction. Singly housed mice were divided into two groups, according to their behavior during the control interaction (vehicle treatment) with the group-housed partner: (a) an aggressive one (attacking group-housed mouse at least once), and (b) a timid one [exhibiting no attacks, displaying defensive-escape (timid) behavior toward the group-housed mouse]. The number, latency, and duration of attacks, tail rattles, unrests (aggressive activities), defenses, escapes, alert postures (timid activities), social sniffing, climbing, and following the partner (sociable behavior) exhibited by singly housed mice were recorded and evaluated by the hardware/software Observer 3.1, Noldus Technology, Holland. Aggressive singly housed mice were subdivided into the two groups – one receiving vehicle (9 mice) and one receiving PG (11 mice). The agonistic interactions with group-housed mice were video-recorded after 14 and 30 days of vehicle/drug administration. The test protocol was adopted from Sulcova and Krsiak (1987).

### Statistical analysis

Data are presented as the group means ± standard error of mean (SEM). Statistical analyses were performed by the program GraphPad Prism 6.0 (San Diego, CA, USA). The statistical significance for the social defeat test was detected by the two-way ANOVA, followed by Sidak’s *post hoc* test. The treatment (two factor level) and the stress (two factor levels) served as independent variables. In the agonistic interaction test, the effect of the treatment (two factor level) and the length of administration (three factor levels) of PG/vehicle were assessed by the two-way repeated measures ANOVA (control interaction first day vs. interactions days 14th and 30th). In the other tests, where the treatment effect was assessed alone, the one-way ANOVA was conducted. Sidak’s *post hoc* test was used when appropriate. The significant level was set at *p* < 0.05.

## Results

### Open field

Statistical analysis of the locomotor activity in the open field by the one-way ANOVA showed significant differences in total distance traveled *F*(6, 40) = 8.034, *p* < 0.0001. MK-801 0.3 mg/kg and ketamine 10 mg/kg induced hyperlocomotion (Figure 1) during 50 min of an exploration. PG plasma levels were highest 15 min after i.p. administration, and brain neurosteroid level peaks occurred 60 min after i.p. application (Rambousek et al., 2011). Therefore, we chose a time interval of 30 min after i.p. application of PG for the subsequent tests.

### Elevated plus maze

**Figure 2:**
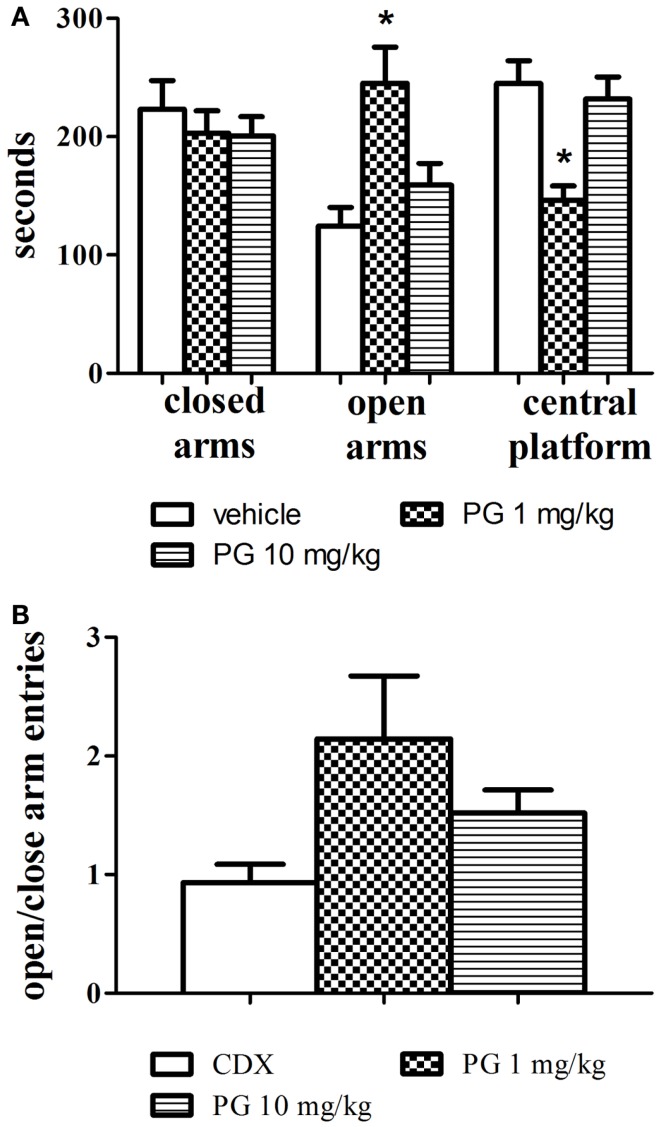
**Anxiolytic effect of PG was assessed in an elevated plus maze**. **(A)** PG administered at the dose of 1 mg/kg significantly increased the time spent in the open arms, and decreased the time spent in the central platform. Mice treated with PG at the dose of 10 mg/kg did not display anxiolytic behavior. All values represent group means ± SEM. *p < 0.005 compared to the vehicle, β-CD group. **(B)** The analysis of open to close arm entries did not reveal differences between treatment groups, probably due to big differences in variance within each group. All values represent group means ± SEM.

One-way ANOVAs revealed that PG administered at the dose of 1 mg/kg significantly increased the time spent in the open arms, *F*(2, 24) = 7.654, *p* = 0.0027 and decreased the time spent in the central platform, *F*(2, 24) = 10.01, *p* = 0.0007. Mice treated with PG 10 mg/kg did not display anxiolytic behavior, and their times spent in the open arms and central platform did not differ from the control group treated with only the vehicle. The treatment had no effect at all on the time spent in the closed arms (Figure 2A). Time spent in the central platform may be indicative of risk assessment behavior. Increased time spent in the central platform may be a sign of hesitation before entering either arm. In order to evaluate locomotor activity in the elevated plus maze, an analysis of open to close arms entries was conducted as well. However, no significant difference was revealed (Figure 2B), probably due to a big variance within each treatment group.

### Forced swimming

**Figure 3:**
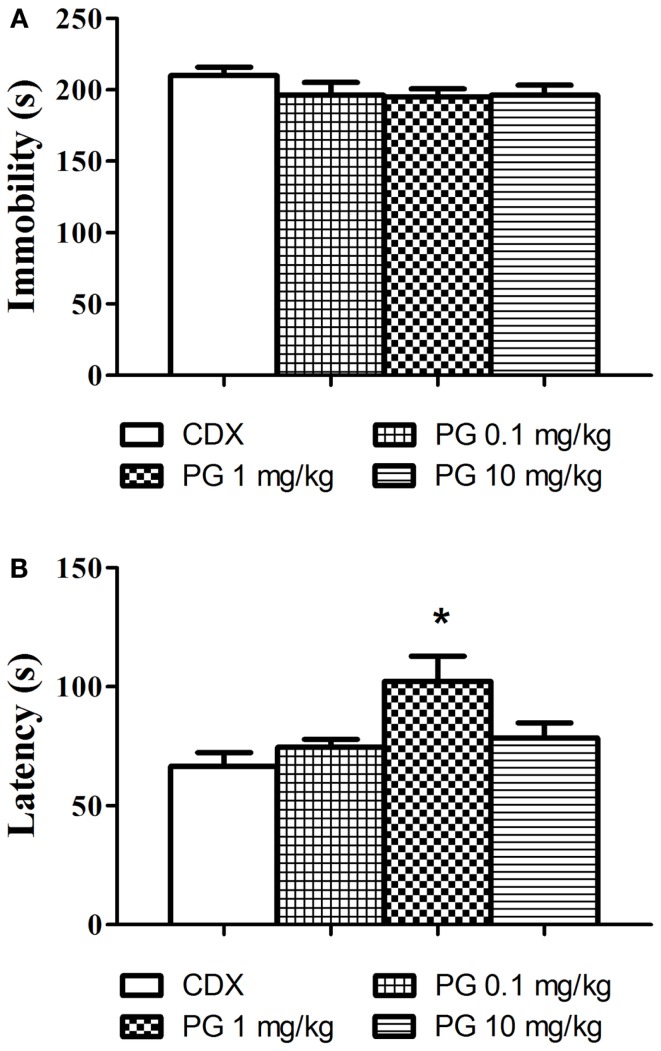
**In the forced swim test, we evaluated the possible antidepressant effect of PG**. **(A)** Immobility time in the forced swim test was assessed 30 min after i.p. administration of PG and vehicle. None of the doses applied significantly influenced floating. Average group values ± SEM. **(B)** In addition to immobility duration in the forced swimming test, we measured time to the first floating as well. Administration of PG at the dose of 1 mg/kg significantly prolonged the latency to the first floating compared to controls. All values represent group means ± SEM. *p < 0.005 compared to the vehicle group.

Even though PG treatment led to the slight decrease in immobility time (Figure 3A), none of the doses used significantly reduced the immobility in comparison with the controls. As the next parameter, we assessed the time of the first floating (latency, Figure 3B). One-way ANOVA detected significant elongation of the latency after PG 1 mg/kg treatment *F*(3, 29) = 5.248, *p* = 0.0051. The discrepancy between results obtained from immobility and the latency analyses remains to be clarified. Sensitivity of the two parameters to antidepressant treatment may differ.

### Social defeat

**Figure 4:**
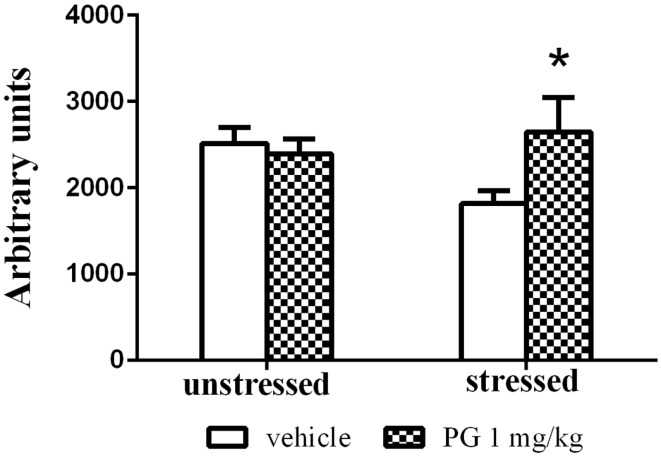
**Group-housed mice repeatedly stressed by defeat on four agonistic interactions (7 days apart) with aggressive singly housed partners exhibited depressed locomotor activity in the open field**. This effect was disinhibited by treatment with PG at the dose of 1 mg/kg. There was no difference in unstressed animals between two treatment groups in the open-field activity. All values represent group means ± SEM. *p < 0.05 compared to the stressed vehicle treated animals.

Group-housed mice repeatedly defeated on aggressive agonistic interactions with singly housed partners exhibited decreased locomotor activity in the open field. Two-way ANOVA revealed significant effect of treatment vs. stress interaction *F*(1, 46) = 4.687, *p* = 0.0356. No significance was found for the effect of factor stress and factor treatment. PG 1 mg/kg administration normalized the stress-induced inhibition of locomotor activity in the open field, expressed as an overall distance traveled (Figure 4).

### Agonistic interactions

**Figure 5:**
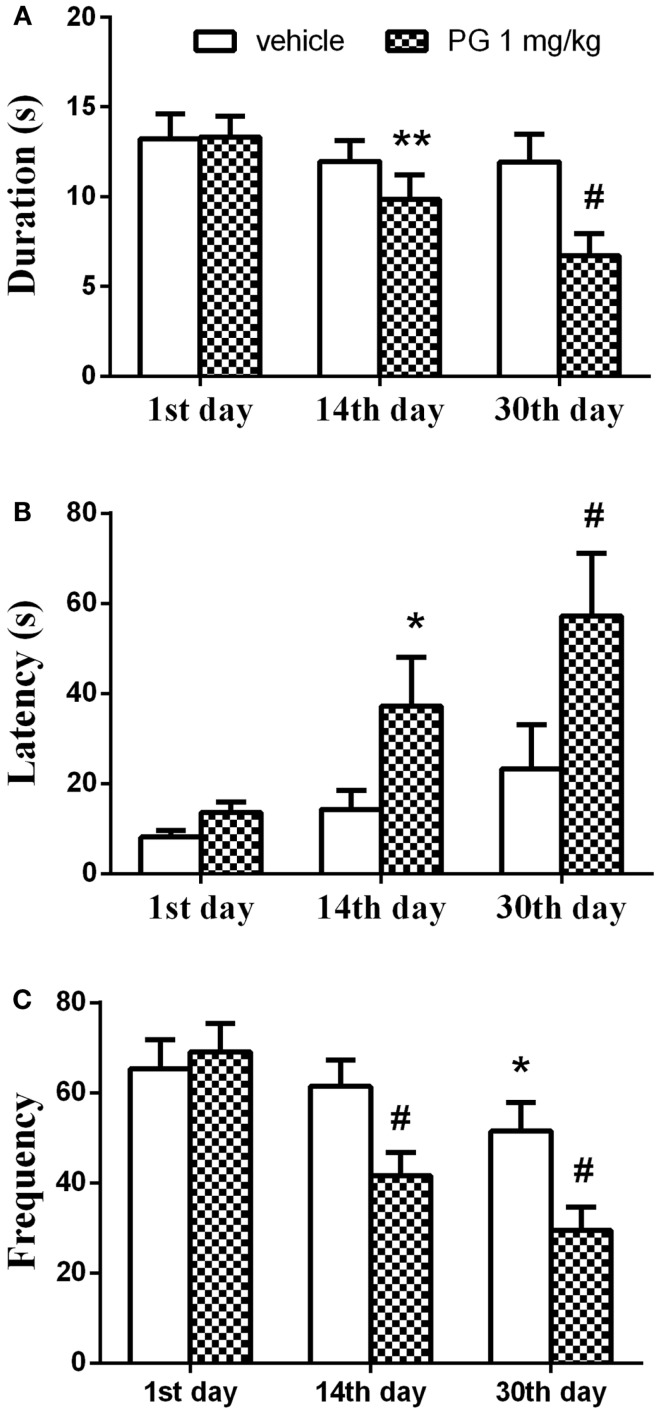
**In 4-min paired agonistic interactions, singly housed mice were confronted with non-aggressive group-housed partners**. Chronic administration of PG at the dose of 1 mg/kg over 30 days reduced aggressive behavior in singly housed mice exhibiting aggressive behavior during the first agonistic interaction. **(A)** Graph shows the duration of aggressive activities. The duration was significantly reduced after 14 and 30 days of PG 1 mg/kg administration, compared to the scores of vehicle treated controls in the first day. The effect was more prominent after 30 days. **(B)** Graph shows latency to the first aggressive action. Latency prolonged in PG treated mice after 14 and 30 days of PG application in comparison to controls. Again this effect was greater after longer administration. **(C)** Graph displays number of aggressive actions. Frequency decreased over the course of the experiment. Vehicle treated mice also showed significant reduction in the frequency of aggressive actions between the 1st and 30th day. However, this change in frequency was not as prominent as in PG treated animals. All values represent group means ± SEM. *p < 0.05, **p < 0.005, ^#^*p* < 0.0001 compared to the vehicle treated animals on the first day of agonistic interaction.

Chronic administration of PG at the dose of 1 mg/kg over 30 days reduced aggressive actions in singly housed mice exhibiting aggressive behavior on the first interaction. Two-way repeated measures ANOVA detected significant effect of the treatment vs. the length of administration interaction in the parameter duration *F*(2, 116) = 5.407, *p* = 0.0057. Length of the administration showed significant effect *F*(2, 116) = 11.97, *p* < 0.0001, whereas treatment showed no influence on the duration of aggression. Aggression was significantly reduced after 14 and 30 days of PG 1 mg/kg administration, compared to the scores of vehicle treated controls in the first day (Figure 5A). This reduction was more pronounced after 30 days of PG treatment (*p* < 0.0001 *post hoc* analysis), compared to only 2 weeks treatment (*p* = 0.0037). The time spent in aggressive interactions dropped in PG treated animals.

Time to the first aggressive action (the latency) was prolonged in PG treated mice. Two-way repeated measures ANOVA revealed the effect of the factor treatment *F*(1,58) = 4.55, *p* = 0.0372 and the factor of administration length *F*(2, 116) = 8.652, *p* = 0.0003 on the latency. Interaction between the two factors was not significant. It took more time for the PG treated animals to attack the intruder after 14 and 30 days of PG application in comparison to the controls (Figure 5B). Again, this effect was greater after longer administration (*p* < 0.0001 after 30 days vs. *p* = 0.0275 after 14 days, revealed by *post hoc* test).

The frequency of aggressive actions decreased over the course of an experiment. Interaction between the two factors, treatment, and length of administration, came out significant [*F*(2, 116) = 8.505, *p* = 0.0004] when analyzed by the two-way repeated measures ANOVA. The length of administration had a significant effect *F*(2, 116) = 30.04, *p* < 0.0001 on the frequency. The kind of treatment we used had no effect. Frequency expressed as a number of aggressive actions decreased after 14 and 30 days of PG treatment. Vehicle treated mice also showed significant reduction in the frequency of aggressive actions between the 1st and 30th day. However, this change in frequency was not as prominent (*p* = 0.0163) as in PG treated animals (*p* < 0.0001 for both time intervals) (Figure 5C). Taken altogether, mice treated daily with PG at the dose of 1 mg/kg over the course of 30 days indulged less in aggressive interactions.

## Discussion

The present study focused on the evaluation of antidepressant-like and anxiolytic-like effects of newly synthesized neurosteroid PG. Neurosteroids are known for their neuroprotective and antipsychotic effects (Pringle et al., 2003; Veiga et al., 2003; MacKenzie et al., 2007; Rambousek et al., 2011; Vales et al., 2012), and alternation of their brain levels is well-documented in various neurodegenerative diseases and aging (Vallée et al., 1997; Nafziger et al., 1998; Kim et al., 2003; Aldred and Mecocci, 2010; Luchetti et al., 2010; Sorwell and Urbanski, 2010). Similarly, the downregulation of neurosteroid synthesis possibly contributes to the development of depressive disorders and anxiety (Morrow, 2007; Schüle et al., 2011, 2014). Neurosteroids as potent NMDAR antagonist and GABA receptor agonists might be promising therapeutic agents in depressive disorders (Zorumski et al., 2013).

Our results from the open-field test indicate that hyperlocomotion found in MK-801 and ketamine treated animals is not present after PG application (Figure 1). It is in concordance with our previous results (Vales et al., 2012). As opposed to non-competitive NMDA antagonists such as dizocilpine (MK-801), ketamine, and PCP often used for induction of schizophrenia-like behavior (Bubeníková-Valesová et al., 2008), PG does not display psychotomimetic properties, but quite the contrary. Administration of PG at the doses of 0.1 and 1 mg/kg did not significantly influence spontaneous locomotor activity in comparison to the control (Figure 1). Furthermore, PG at the highest dose of 10 mg/kg exhibited hypolocomotion after i.p. administration. It cannot be interpreted as an unexpected effect, because GABA agonists as well as NMDA antagonists are drugs, which produce typically sedative and anesthetic effects.

The lower risk of hyperlocomotion can be explained by the different mechanism of action. PG is a use-dependent NMDA inhibitor, which has a more pronounced inhibitory action on the tonically active NMDAR (Petrovic et al., 2005). The hypothesis underlying the ability of use-dependent inhibitors to differentiate between phasic physiological and tonic pathological activation of NMDAR during pathological states have gained relatively wide acceptance. However, it is still unclear how such compounds could differentiate between normal and abnormal synaptic activation of NMDAR (Borovska et al., 2012). The lowered risk of hyperlocomotion displays memantine as well. Memantine has been shown to result in the preferential blockade of excessive NMDAR activity, while sparing normal excitatory synaptic function (Lipton, 2006, 2007).

Anxiolytic performance of PG was assessed in the elevated plus maze test. Agonists of GABA receptors are recognized for their anxiolytic properties (Brot et al., 1997), and in the elevated plus maze test they increase the time spent in open arms (Rodgers and Johnson, 1998). PG at the dose of 1 mg/kg exhibited anxiolytic-like activity clearly indicated by significant increase in time spent in open arms (Figure 2A). Anxiolytic-like properties of PG were also previously confirmed by decreased shock-induced ultrasonic vocalization in rats after PG application (Vales et al., 2012). The analysis of open to close arm entries did not reveal differences between treatment groups (Figure 2B), however the decrease in locomotion in the PG of 10 mg/kg treated group was not as prominent as in the open field. It can be explained by the decreased habituation to the maze, since animals spent considerably less time in the elevated plus maze than in the open field, and the activity of the mice is enforced by the nature of the task.

Pregnenolone glutamate at all doses slightly reduced floating when applied 30 min prior to the test, however this decrease was not significant (Figure 3A). We analyzed not only the overall time of floating in the session, but also the time to the first floating. In our experiment, PG 1 mg/kg significantly prolonged the latency to the first immobility (Figure 3B). Time to the first floating analysis produced more robust results, at least in our experimental set-up. According to literature available and our experience, the forced swim test is sensitive to various independent variables, such as mouse strains (Lucki et al., 2001; Mason et al., 2009), age of mice used (Mason et al., 2009; Sequeira-Cordero et al., 2013), water temperature (Pintér et al., 2011), 2 days vs. 1 day protocol, water depth (Pintér et al., 2011), diameter of cylinders animals swim in, seasonal changes, etc. (Petit-Demouliere et al., 2005). We probably failed to find the optimal protocol where the differences between the groups would be more distinct.

3α-reduced neuroactive steroids have anxiolytic and antidepressant-like effects in the preclinical studies (Eser et al., 2006). Even though PG meets this effect, it failed to improve immobility scores in the forced swim test. Similar results were obtained after progesterone application (Urani et al., 2001). However, the administration of allopregnanolone significantly reduced the time spent by immobilization (Rodríguez-Landa et al., 2009), indicating that allopregnanolone is a more potent GABA activator, compared to progesterone. The mechanism of action of PG on GABA neurons is not fully known. If the failure to decrease immobility in the forced swimming could be ascribed to the lower affinity of PG to GABA receptors, or if it is caused by the methodological drawbacks in the test, remains to be clarified.

Pregnenolone glutamate at the particular dose of 1 mg/kg had the most pronounced effect in the tests mentioned above. Therefore, we decided to use this concentration in the repeated social defeat model. This animal model of depressive disorder is more plausible than the forced swim test, since it mimics a different aspect of depression by prolonged exposure to psychosocial stress (Chaouloff, 2013; Venzala et al., 2013). In the social defeat test, PG at 1 mg/kg normalized the locomotor activity in the open field in mice exposed to aggressive conspecific prior to open-field testing (Figure 4). Stressed mice receiving no PG exhibited stress-induced reduction in locomotion measured by distance passed. There was no difference in unstressed animals between the two treatment groups in the open-field activity. The spontaneous locomotor activity was not affected by PG administration; this conclusion is also supported by the results from the open field.

The social defeat stress leads to depression-like abnormalities in defeated animals, lasting for weeks, such as anxiety, social avoidance, anhedonia, and changes in body weight, increased blood pressure, suppressed immune responses, and others (Blanchard et al., 2001; Huhman, 2006; Chaouloff, 2013). Among the most often noted behavioral responses is the decrease in activity and exploration in the open field (Meerlo et al., 1996; Rygula et al., 2005; Razzoli et al., 2009). Chronic, but not acute, treatment with antidepressants, both SSRI and tricyclics, can reverse the consequences of social stress exposure (van Bokhoven et al., 2011; Olivares et al., 2012; Venzala et al., 2012). Similarly, NMDAR antagonist (Jasnow et al., 2004) and GABA receptor agonist (Jasnow and Huhman, 2001) microinjected to the amygdala block conditioned defeat.

Aggressive mice after 30 days of single-housing were receiving PG of 1 mg/kg chronically over 30 days. PG administration led to the significant decrease of aggressive behavior in the agonistic interactions with non-aggressive group-housed partners, when compared to the control interaction in the first day of the experiment. The duration and frequency of the aggressive behavior decreased after 14 days of chronic application of PG. An even more profound effect was reached after 30 days of application (Figures 5A,C). The significant decrease in frequency was also detected in vehicle treated animals 30 days after β-CD administration. However, this effect was less prominent, compared to PG treated animals (Figure 5C), and it might be caused by habituation to intruder. The latency to the first aggressive action was prolonged 14 days after PG administration, and it was again accompanied by a more profound effect 30 days after (Figure 5B). Taken together, the mice treated daily with PG at the dose level of 1 mg/kg over the course of 30 days indulged less in aggressive interactions.

Stress induced by social isolation causes a significant decrease in pregnenolone, progesterone, allotetrahydrodeoxycorticosterone, and allopregnanolone concentrations in the cerebral cortex, compared to the group-housed controls (Serra et al., 2000). The neuroactive steroid changes were not evident after 48 h of chronic stress exposure, but their decrease was present 7 days after the chronic stress, with the most prominent change 30 days after (Serra et al., 2000). Stress and depression are associated with a decrease in GABAergic function in the PFC and hippocampus (Benes et al., 2008; Croarkin et al., 2011). Administration of PG could lead to normalization of the level of GABA potentiating neurosteroids, and therefore contribute to the homeostasis restoration (aggression reduction/normal locomotor activity) by enhancement of the GABA neurotransmission.

Chronic stress reduces the production of allopregnanolone, as well as other GABAergic neurosteroids (Serra et al., 2000). Decreased levels of neurosteroids in the plasma and CSF are found in patients with major depression (Romeo et al., 1998; Uzunova et al., 1998). Prolonged exposure to stress may induce a reduction in pituitary responsiveness to high concentrations of CRH, leading to desensitization, and consequently to decreased ACTH secretion (Hoffman et al., 1985), affecting neurosteroid synthesis. A reduced ACTH response to chronic stress leads to hyper-responsiveness of the hypothalamic–pituitary–adrenal axis (HPA) axis to the new stimuli, and confers vulnerability to mood and anxiety-related disorders, as well as depression (Biggio and Purdy, 2001). Antidepressant treatment normalizes the altered allopregnanolone levels (Uzunov et al., 1996; Uzunova et al., 1998; Schüle et al., 2014) suggesting that GABAergic neurotransmission alteration by the neurosteroids has a therapeutic effect.

The increasing number of evidence shows that the NMDARs play a crucial role in the neurobiology and treatment of depression. PG as the use-dependent inhibitor of NMDARs binds only to the extrasynaptic and tonically activated NMDARs, leaving normal neurotransmission unaffected (Rambousek et al., 2011), and therefore causing less severe side-effects. The effect of neuroactive steroids appears to be subtype selective (Gibbs et al., 2006). Similarly, PG displays more potent inhibition of responses mediated by NR1/NR2C-D receptors, compared to those mediated by NR1/NR2A-B receptors (Petrovic et al., 2005). The current challenge in development of steroidal NMDA antagonists suitable for clinical use is in the low potency of existing drugs. Nonetheless, if both the GABA_A_ enhancers and NMDA antagonists have antidepressant potential, the ideal agent might be the one that combines all these effects in a single molecule (Zorumski et al., 2013). Therefore, it might be surprising that DHEA and DHEAS as positive NMDA and negative GABA_A_ modulators exert antidepressant activity (Wolkowitz et al., 1997, 1999; Urani et al., 2001). However, the mechanism of action of DHEA/S suggests an indirect way of normalizing HPA axis activity (via MAP2C protein activity, serotonin turn-over, anti-glucocorticoid effects, MAO inhibitory effect, promotion of neurogenesis, etc.) (Maninger et al., 2009; Pérez-Neri et al., 2009; Felice et al., 2012).

Taken altogether, these results showed that 3α5β-PG activity at NMDARs lacks negative side-effects accompanying treatment with non-competitive NMDA antagonists that impair normal neurotransmission. Since PG combines both effects – GABA_A_ receptor potentiation and the NMDAR inhibition – it has a potential as an antidepressant in the treatment of depressive symptoms.

In conclusion, we demonstrated in animal models antidepressant-like and anxiolytic-like activities of 3α5β-PG – an analog of naturally occurring 3α5β-pregnanolone sulfate. PG is an example of a promising neurosteroid showing possible potential for development of a novel antidepressant, procognitive, and neuroprotective agents. This branch of research gives rise to a possibility of obtaining drugs with antidepressant/anxiolytic properties and minimal side-effects, i.e., with a more favorable risk/benefit ratio.

## Conflict of Interest Statement

The authors declare that the research was conducted in the absence of any commercial or financial relationships that could be construed as a potential conflict of interest.

## Abbreviations

ACTH, adrenocorticotropic hormone; β-CD, hydroxypropyl-β-cyclodextrin; CNS, central nervous system; CRH, corticotropin-releasing hormone; DHEA/S, dehydroepiandrosterone sulfate; GABA, γ-aminobutyric acid; HPA axis, hypothalamic–pituitary–adrenal axis; MK-801, dizocilpine; NMDA, *N*-methyl-d-aspartic acid; NMDAR, *N*-methyl-d-aspartic acid receptor; PG, 3α5β-pregnanolone glutamate; SSRI, selective serotonin reuptake inhibitor.
